# Intestinal Delivery of Proinsulin and IL-10 via *Lactococcus lactis* Combined With Low-Dose Anti-CD3 Restores Tolerance Outside the Window of Acute Type 1 Diabetes Diagnosis

**DOI:** 10.3389/fimmu.2020.01103

**Published:** 2020-06-09

**Authors:** Dana P. Cook, João Paulo Monteiro Carvalho Mori Cunha, Pieter-Jan Martens, Gabriele Sassi, Francesca Mancarella, Giuliana Ventriglia, Guido Sebastiani, An-Sofie Vanherwegen, Mark A. Atkinson, Karolien Van Huynegem, Lothar Steidler, Silvia Caluwaerts, Pieter Rottiers, Luc Teyton, Francesco Dotta, Conny Gysemans, Chantal Mathieu

**Affiliations:** ^1^Clinical and Experimental Endocrinology (CEE), Department of Chronic Diseases, Metabolism and Ageing, Campus Gasthuisberg O&N 1, Katholieke Universiteit Leuven (KU Leuven), Leuven, Belgium; ^2^Diabetes Unit, Department of Medicine, Surgery and Neurosciences, University of Siena and Fondazione Umberto Di Mario ONLUS—Toscana Life Science Park, Siena, Italy; ^3^Immunology and Laboratory Medicine, Department of Pathology, College of Medicine, University of Florida Diabetes Institute, Gainesville, FL, United States; ^4^ActoBio Therapeutics™, Ghent, Belgium; ^5^The Teyton Lab, Department of Immunology and Microbiology, Scripps Research Institute, La Jolla, CA, United States

**Keywords:** type 1 diabetes, antigen-specific therapy, anti-CD3, genetically modified *Lactococcus lactis*, beta cell mass, oral tolerance

## Abstract

A combination treatment (CT) of proinsulin and IL-10 orally delivered via genetically modified *Lactococcus lactis* bacteria combined with low-dose anti-CD3 (aCD3) therapy successfully restores glucose homeostasis in newly diagnosed non-obese diabetic (NOD) mice. Tolerance is accompanied by the accumulation of Foxp3^+^ regulatory T cells (Tregs) in the pancreas. To test the potential of this therapy outside the window of acute diabetes diagnosis, we substituted autoimmune diabetic mice, with disease duration varying between 4 and 53 days, with syngeneic islets at the time of therapy initiation. Untreated islet recipients consistently showed disease recurrence after 8.2 ± 0.7 days, while 32% of aCD3-treated and 48% of CT-treated mice remained normoglycemic until 6 weeks after therapy initiation (*P* < 0.001 vs. untreated controls for both treatments, *P* < 0.05 CT vs. aCD3 therapy). However, mice that were diabetic for more than 2 weeks before treatment initiation were less efficient at maintaining normoglycemia than those treated within 2 weeks of diabetes diagnosis, particularly in the aCD3-treated group. The complete elimination of endogenous beta cell mass with alloxan at the time of diabetes diagnosis pointed toward the significance of continuous feeding of the islet antigen proinsulin at the time of aCD3 therapy for treatment success. The CT providing proinsulin protected 69% of mice, compared to 33% when an irrelevant antigen (ovalbumin) was combined with aCD3 therapy, or to 27% with aCD3 therapy alone. Sustained tolerance was accompanied with a reduction of IGRP^+^CD8^+^ autoreactive T cells and an increase in insulin-reactive (InsB12–20 or InsB13–2) Foxp3^+^CD4^+^ Tregs, with a specific accumulation of Foxp3^+^ Tregs around the insulin-containing islet grafts after CT with proinsulin. The combination of proinsulin and IL-10 via oral *Lactococcus lactis* with low-dose aCD3 therapy can restore tolerance to beta cells in autoimmune diabetic mice, also when therapy is started outside the window of acute diabetes diagnosis, providing persistence of insulin-containing islets or prolonged beta cell function.

## Introduction

Aberrant recognition of self-antigen(s) by T cells can cause autoimmune diseases such as type 1 diabetes (T1D). The pursuit for a cure of T1D through restoration of immune tolerance for the pancreatic insulin-producing beta cells is ongoing and encouraging observations have been made in animal models of the disease with some hints of success in humans. Promising interventions include CTLA4-Ig (Abatacept), anti-thymocyte globulin (ATG), and high-dose Fc receptor (FcR) non-binding CD3-specific monoclonal antibody (Teplizumab) therapy (1–3). However, at present, none of these approaches has reached the clinical practice, as the balance between potential benefits (e.g., restoration of normoglycemia without exogenous insulin dependence) and long-term side effects (e.g., cytokine release and serum sickness syndromes, Epstein-Barr virus reactivation, high infection rates, and systemic immune suppression) is considered unfavorable. Combination therapies using lower doses of highly targetspecific drugs or combinations of low-dose immune modulation therapy and antigen introduced via tolerogenic routes are appealing strategies that are currently being explored in clinical trials (ClinicalTrials.gov Identifier: NCT03751007; NCT02620332, NCT02837094).

Most clinical trials focus on newly diagnosed T1D patients, in whom the residual beta cell mass is presumably sufficient to achieve restoration of normoglycemia, provided that the immune system can be arrested in its attack of the beta cells. It is estimated that newly diagnosed T1D patients have a remaining functional beta cell mass of around 25–40% of its full capacity (3, 4), but many patients maintain beta cell function for several years after diagnosis (5–7). However, the presence of beta cell mass may not be the only argument making interventions in newly diagnosed T1D patients a sweet spot. In animal models, there appears to be a difference in therapeutic efficacy when interventions are applied at the time of diabetes diagnosis, compared to moments outside of this critical window of opportunity, independent of functional beta cell mass (8). In addition, the stage of the autoimmune response may potentially affect therapeutic outcome. As such, anti-CD3 (aCD3) monoclonal antibody therapy is most efficacious when applied at diabetes diagnosis (9).

An interesting approach proposed in recent years is the combination treatment (CT) of low-dose aCD3 therapy with the whole islet antigen proinsulin (PINS) and the pro-tolerogenic cytokine IL-10 administered orally via genetically modified *Lactococcus lactis* (*L. lactis*) bacteria (10, 11). This targeted therapeutic approach consistently showed T1D reversal in around 60% of newly diagnosed non-obese diabetic (NOD) mice. The intervention was accompanied by the accumulation of Foxp3^+^ regulatory T cells (Tregs) in the pancreas (10). This therapy was started in NOD mice on the day of diabetes onset, raising the question of whether this therapy is only applicable around the time of diagnosis or whether the restoration of immune tolerance against the beta cells may also occur at later disease stages when functional beta cell mass further deteriorates and antigen and epitope spreading ensues. Antigen-specific approaches that depend on the induction of immune regulation may be successful in reinstating active immune tolerance toward a broader antigen repertoire due to infectious tolerance and bystander suppression (12). In the present study, we designed experiments wherein beta cell mass was substituted by syngeneic islet transplantation in NOD mice with long-duration T1D. We demonstrated that the antigen-based CT was also efficacious outside the window of acute diabetes diagnosis. Success was linked to disease duration, but also to the delivery of a disease-relevant antigen, in this case PINS, which was needed for Foxp3^+^ Tregs to become insulin-reactive and home preferentially to insulin-containing islet grafts.

## Methods

### Animals

NOD mice were inbred in KULeuven animal facility since 1989 and kept under semi-barrier conditions. Mice were screened 3 times a week for glucosuria and consequently considered diabetic if non-fasting blood glucose concentrations exceeded 200 mg/dL for 2 consecutive days (AccuCheck, F. Hoffmann-La Roche Ltd., Basel, Switzerland). Mice were bred and housed according to protocols approved by the KULeuven Animal Care and Use Committee (Leuven, Belgium; project number 116/2015), and experiments complied with the EU Directive 2010/63/EU for animal experiments.

### *L. lactis* Culture

Genetically modified *L. lactis* bacteria secreting the whole human PINS antigen and human IL-10 or chicken ovalbumin and human IL-10 (LL-OVA) were generated by ActoBio Therapeutics™ (Zwijnaarde (Ghent), Belgium) and grown as described (10, 13). For oral administration, stock suspensions were diluted 1,000-fold in growth medium and incubated for 16 h at 30°C, reaching a saturation density of 2 × 10^9^ colony forming units (CFU)/mL. Bacteria were harvested by centrifugation and concentrated 10-fold in BM9 medium. The treatment dose consisted of 100 μL of this concentrated suspension.

### Islet Isolation and Transplantation

Islets were isolated from 2- to 3-week-old insulitis-free NOD mice as described (14). For beta cell substitution, 500 freshly isolated islets were grafted beneath the left kidney capsule of autoimmune diabetic mice (between 4 and 53 days after diabetes diagnosis). Transplantation was considered successful if the non-fasting blood glucose concentration returned to normal (<200 mg/dL) within 24 h after surgery. Weight and blood glucose concentrations from the tail vein were monitored three times a week after transplantation with an AccuCheck glucometer. Graft survival time was calculated as the number of days before disease recurrence. Mice that remained normoglycemic until 6 weeks after transplantation were considered cured. For non-cured mice, the day of disease recurrence was defined as the first of 2 consecutive days of non-fasting blood glucose concentrations >250 mg/mL. Only mice being diabetic for more than 2 weeks before islet substitution were used for metabolic, histological, and flow cytometric analysis. Experiments were performed at 10 days (when all mice were still normoglycemic), at 6 weeks after treatment initiation [when normoglycemia was maintained (cured)] or at the time of disease recurrence (untreated and non-cured). All mice were sacrificed according to humane end-points (i.e., 20% weight loss or three consecutive maximum blood glucose measurements >600 mg/dL).

### Treatment Groups

Newly diagnosed diabetic mice were kept under insulin pellets (Linbit™; LinShin Canada, Inc., Ontario, Canada) for up to 53 days post-diagnosis, which constituted long-duration T1D mice. Then, all mice received a transplantation of 500 insulitis-free syngeneic islets (day 0) and were further left untreated (control, CTRL), or entered into one of two treatment arms: hamster anti-mouse CD3 antibody (clone 145-2C11, BioXCell, West Lebanon, NH) was administered intravenously (2.5 μg/d; total of 12.5 μg) for 5 consecutive days alone (aCD3), or combined with oral administration of *L. lactis* bacteria secreting PINS with IL-10 (2 × 10^9^ CFU/d) 5 times per week for a period of 6 weeks (CT) (Supplementary Figure 1A). In a separate cohort, newly diagnosed diabetic NOD mice were injected with alloxan (90 mg/kg i.v., Sigma) in order to completely deplete residual endogenous beta cell mass. After 48 h, all mice received 500 insulitis-free syngeneic islets and were either (1) left untreated (CTRL), (2) treated with aCD3 alone (aCD3), (3) aCD3 combined with *L. lactis* bacteria secreting PINS with IL-10 (CT), or (4) aCD3 combined with *L. lactis* secreting a non-islet antigen, ovalbumin with IL-10 (aCD3+LLOVA) (Supplementary Figure 1B).

### Metabolic Beta Cell Function

Random C-peptide concentrations in heparinized plasma were measured by ELISA (Merck Millipore). Pancreases and kidneys were harvested for histological analyses and/or for insulin content determination by ELISA (Mercodia, Uppsala, Sweden) as described (10).

### Histology and Confocal Immunofluorescence

Islet graft-bearing kidneys were fixed in 4% buffered formalin and embedded in paraffin at the time of diabetes recurrence or at selected time points after islet implantation. Sections were stained with hematoxylin and eosin (H&E). Insulin staining was performed as previously described (10). Beta cell mass of the islet grafts was determined by Z-stack confocal microscopy analysis, capturing five different focal planes from each graft, and then quantifying the insulin^+^ volume (μm^3^) using Volocity 6.3 software (Perkin Elmer, Waltham, MA). Foxp3 staining was performed as described (10) and Foxp3^+^ Treg enumeration was performed by manual cell counting.

Immunofluorescence imaging on graft-bearing kidneys snap-frozen in Tissue-Tek OCT compound (Sakura Fineteck, Torrance, CA) was used to visualize immune populations in the graft. Sections were stained using antibodies directed against CD4 (clone RM4-5; #550280, BD Biosciences, Erembodegem, Belgium) or CD8a (clone 53-6.7; #550281, BD Biosciences), followed by AF488-conjugated goat anti-rat IgG as a secondary antibody.

### Flow Cytometry Analysis

Single-cell suspensions were prepared from spleen, kidney and kidney draining lymph nodes (KLN) by mechanical disruption. Cells were washed with fluorescence-activated cell sorting (FACS) buffer [phosphate-buffered saline (PBS) containing 3% FBS and 0.05% NaN3] and incubated with avidin (0.5 mg/ml; Sigma-Aldrich, St. Louis, MI) and Fc block (BD Biosciences) in FACS buffer for 1 h at room temperature. Thereafter, cells were stained with directly conjugated antibodies against CD4, CD8α, CD25, CD44, CD62L, B220, F4/80 (eBioscience, Thermo Fisher Scientific). Intracellular staining for Foxp3 was also performed according to manufacturer's instructions using a Foxp3 staining kit (eBioscience, Thermo Fisher Scientific). Insulin-reactive (e.g., InsB12–20 (TEGVEALYLVC-GGGS) and InsB13–21 (TEGEALYLVCGEGGS) Foxp3^+^CD4^+^ T cells were detected using PE- and APC-labeled MHC/peptide tetramers (tet), used at a final concentration of 10 mg/ml in FACS buffer for 1 h at room temperature (kind gift by Luc Teyton, Scripps Research Institute, La Jolla, Ca) (15). Gates were set on FSC^int^ SSC^int^ (lymphocytes), live (Zombie Aqua™, Biolegend, San Diego, CA), Lin^−^ (CD8, F4/80, B220), single cells (FSCA/FSC-H), CD4^+^, CD25^+^ and Foxp3^+^. Values indicate the absolute number of tetramer positive (tet^+^) InsB12–20 or InsB13–21 cells per 100 Foxp3^+^ Tregs, either CD25^+^ or CD25^−^ (Supplementary Figure 2). Absolute numbers were obtained by including CountBright™ absolute counting beads following the manufacturer's instructions (Thermo Fisher Scientific). Frequencies of IGRP-reactive CD8^+^ T cells were detected using NRP-V7 (KYNKANVFL): H-2K^d^ pentamer reagent (ProImmune, Oxford, UK) following manufacturer's instructions. Samples (>100,000 cells) were acquired on a BD Canto II flow cytometer (BD Biosciences), and data were analyzed with FlowJo software (Tree Star Inc., Ashland, OR).

### Statistics

Statistical analyses were performed with GraphPad Prism software (La Jolla, San Diego, CA). KaplanMeier analysis was performed for diabetes-free survival determination, and differences were assessed with a Log-rank (Mantel-Cox) test. All measurement data was presented as the mean ± SEM, unless stated otherwise. Flow cytometry, ELISA, insulin content, and Foxp3^+^ density data were analyzed by either a Mann-Whitney *U* test or a Kruskal-Wallis test with Dunn's correction. Significance was defined as ^*^P < 0.05, ^**^P < 0.01, ^***^P < 0.001, ^****^P < 0.0001.

## Results

### Combination Treatment Efficiently Modulates Beta Cell Autoimmunity in Longstanding Diabetic Mice After Beta Cell Substitution, Depending on Disease Duration and Presence of Islet Antigen

**Figure 1:**
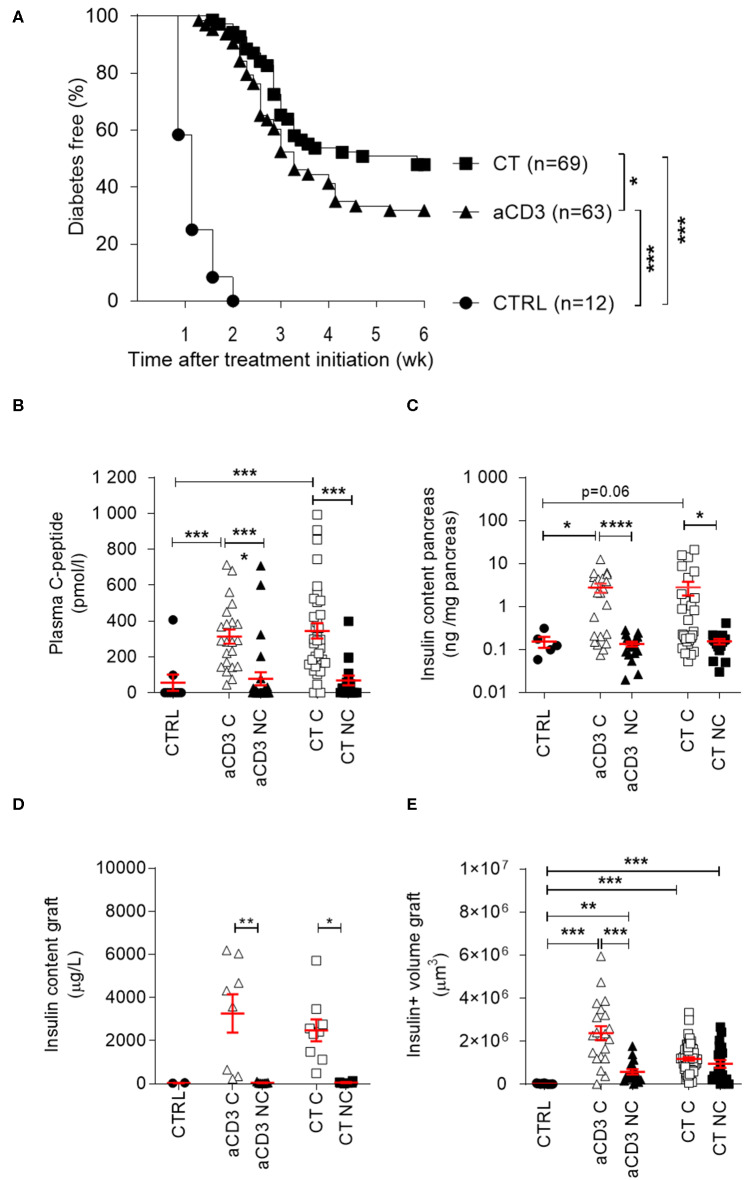
Combination treatment efficiently modulates beta cell autoimmunity in long-duration diabetic mice after islet substitution. NOD mice, being diabetic between 4 and 53 days, were maintained on insulin pellets until islet implantation. At that stage, mice were left untreated (CTRL, *n* = 12) or given a short-term low-dose aCD3 therapy (aCD3, *n* = 63) either alone or combined with *L. lactis* bacteria secreting PINS with IL-10 (CT, *n* = 69). **(A)** Shown is the percentage of mice remaining normoglycemic 6 weeks after therapy initiation as Kaplan-Meier survival curves. Plasma C-peptide concentration **(B)**, insulin content of pancreas **(C)** and graft **(D)**, and insulin (ins)+ volume of graft **(E)** were determined 6 weeks after therapy initiation. Symbols represent individual mice, and line and error bars reflect group mean ± SEM. Open symbols = cured [C], filled symbols = non-cured [NC]. Kruskal-Wallis test followed by Dunn's multiple testing was used for statistical analysis in **(B–E)** and a log-rank test was used in **(A)** *P < 0.01, **P < 0.01, ***P < 0.001, ****P < 0.0001.

To study the efficacy of antigen-specific CT in mice with long-duration T1D (between 4 and 53 days after diabetes onset) and thus, beyond the window of new diagnosis (<2 days after onset) which was previously studied (10), we transplanted 500 insulitis-free syngeneic islets under the kidney capsule at therapy initiation, bringing all mice to the same level of metabolic control (see regimen in Supplementary Figure 1A). If left untreated, hyperglycemia returned within 2 weeks in all mice, with a mean time to disease recurrence of 8.2 (± 0.7) days. Low-dose aCD3 (145-2C11) therapy maintained normoglycemia in 32% of the syngeneic islet recipients until 6 weeks after therapy initiation, with a mean time to disease recurrence of 20 (± 0.9) days. When aCD3 was combined with oral gavage of *L. lactis* bacteria secreting PINS with IL-10, 48% remained free of disease recurrence with a mean survival time of 21.3 (± 1.1) days (Figure 1A). Mice progressing to disease recurrence after aCD3 therapy or CT did so significantly later than untreated controls (Figure 1A). Therapeutic success was reflected by higher random plasma C-peptide concentrations (Figure 1B), higher endogenous beta cell mass (Figure 1C), and higher insulin content/volume in the islet grafts (Figures 1D,E) after 6 weeks.

**Figure 2:**
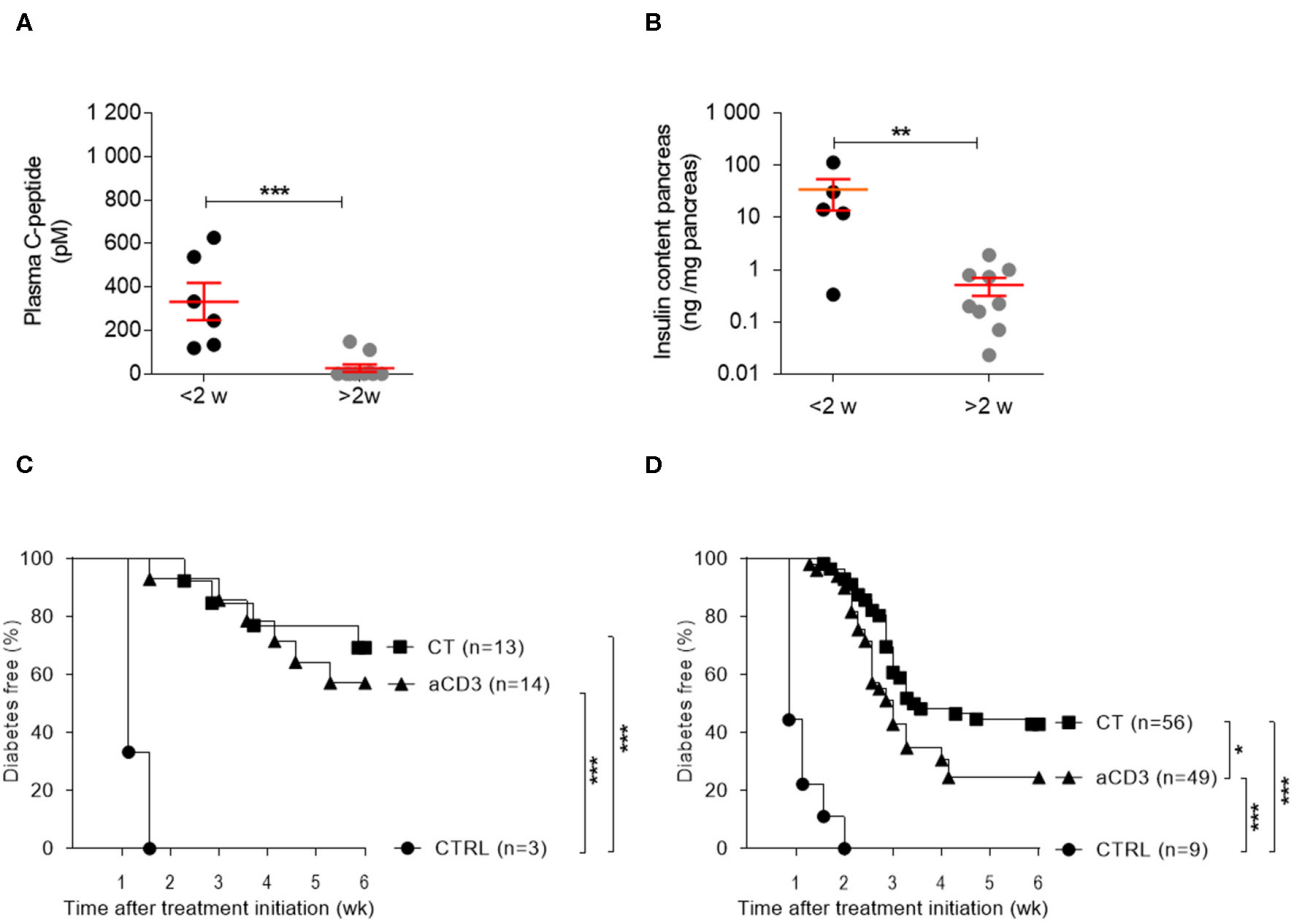
Disease duration determines therapeutic success of the combination treatment in isletsubstituted mice. Disease duration is accompanied by a decline in (functional) beta cell mass and therapeutic outcome. **(A)** Plasma C-peptide concentrations and **(B)** insulin content of pancreas were measured in diabetic NOD mice with disease duration of less (black) or more (gray) than 2 weeks. Symbols represent individual mice, and line and error bars reflect group mean ± SEM. NOD mice, being diabetic for less **(C)** or more **(D)** than 2 weeks, were transplanted with syngeneic islets and were left untreated [CTRL; *n* = 3 **(C)** and *n* = 9 **(D)**] or given a short-term low-dose aCD3 therapy (aCD3; *n* = 14 **(C)** and *n* = 49 **(D)**] either alone or combined with *L. lactis* bacteria secreting PINS with IL-10 [CT; *n* = 13 **(C)** and *n* = 56 **(D)**]. Shown is the percentage of mice remaining normoglycemic after 6 weeks of therapy initiation. Mann-Whitney *U* test was used for statistical analysis in **(A,B)** and a log-rank test was used in **(C,D)**; *P < 0.01, **P < 0.01, ***P < 0.001.

Random plasma C-peptide concentrations and pancreatic insulin content at therapy initiation revealed significant heterogeneity in autoimmune diabetic mice, depending on disease duration. From 2 weeks after diabetes diagnosis, mice typically lacked any measurable sign of (functional) beta cell mass (Figures 2A,B). Both aCD3 therapy and CT were superior at maintaining normoglycemia after islet substitution when mice were diabetic for less, compared to more, than 2 weeks (69 vs. 57% and 43 vs. 24%, respectively), with the CT being more effective than aCD3 therapy (Figures 2C,D). These observations also imply that therapy-cured mice diabetic for more than 2 weeks before transplantation and therapy relied exclusively on their newly implanted islets for glucose control. Untreated controls showed comparable time to disease recurrence, irrespective of disease duration (mean 9 ± 1 vs. 7.8 ± 0.9 days for mice transplanted <2 or >2 weeks after onset, respectively). These results establish that CT efficiently controls disease recurrence in NOD mice receiving syngeneic islets; however, efficacy depends on disease duration.

### Combination Treatment Promotes Islet Graft Survival Despite Massive Immune Infiltration

In order to investigate the protective effect of the antigen-specific CT on beta cell destruction, we examined the islet grafts from NOD recipients after 6 weeks or around the time of disease recurrence. Without therapy, islet grafts were completely destroyed and few intact insulin^+^ islets were detected (data not shown). In contrast, the islet graft structure in NOD recipients cured by both therapies was preserved (Supplementary Figure 3). Immunofluorescent analysis of CD4^+^ and CD8^+^ T cells showed analogous mononuclear accumulation around the grafted tissue in both treatment groups; however, immune cells did not infiltrate into the central area of the islets (Supplementary Figure 3).

### Combination Treatment Eliminates IGRP^+^CD8^+^ Autoreactive T Cells and Increases Foxp3^+^CD4^+^ Tregs in the Islet Graft

A common theme emerging from studies of oral antigen-specific immune tolerance is the elimination of CD8^+^ autoreactive T cells and/or the activation of CD4^+^ Tregs specific for the administered antigen (16). To identify tolerance mechanisms, we studied the downstream cellular events in islet-substituted diabetic NOD mice at both 10 days and 6 weeks after therapy initiation (cured) or at disease recurrence (untreated and non-cured) by flow cytometry analysis. The circulating (splenic) frequencies of naïve, effector memory and central memory CD4^+^ (Supplementary Figure 4A) and CD8^+^ (Supplementary Figure 4B) T cells were not significantly altered with respect to therapeutic success or failure (Supplementary Figure 4), indicating that the CT did not induce general immune suppression and still allowed recognition of foreign antigens.

**Figure 3:**
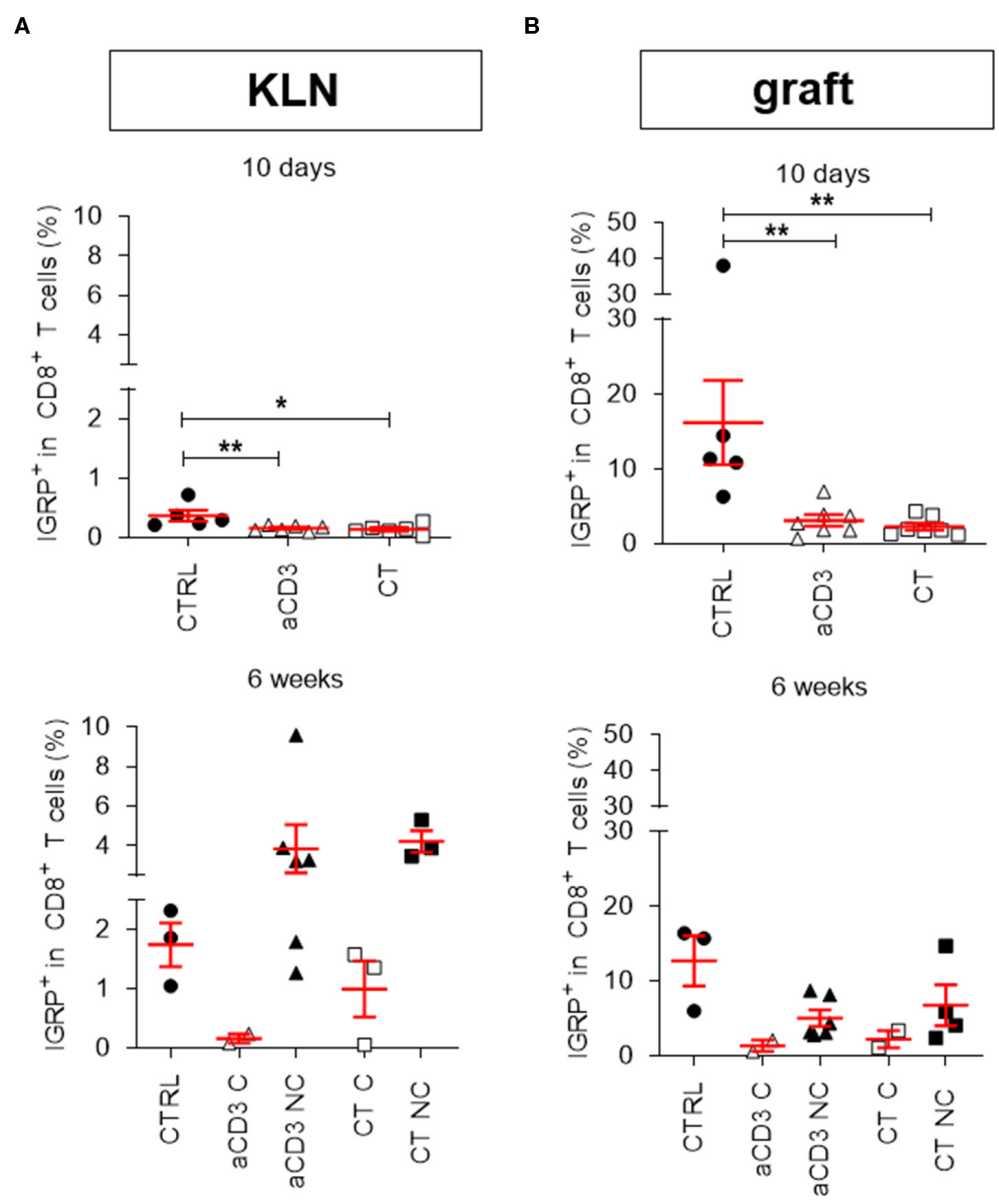
Combination treatment eliminates IGRP^+^CD8^+^ autoreactive T cells in the islet grafts. Frequency of IGRP^+^ cells within CD8^+^ T cell gate is shown at both 10 days and 6 weeks after islet substitution and therapy initiation (cured) or at disease recurrence (untreated and non-cured) in the kidney draining lymph nodes (KLN) **(A)** and islet grafts **(B)** of diabetic NOD mice. Mice were left untreated (CTRL) or given a short-term low-dose aCD3 therapy (aCD3) either alone or combined with *L. lactis* bacteria secreting PINS with IL-10 (CT). Symbols represent individual mice, and line and error bars reflect group mean ± SEM. Open symbols = cured [C], filled symbols = non-cured [NC]. Statistical significance between groups was calculated by Mann-Whitney *U* test; *P < 0.05, **P < 0.01.

The most prevalent CD8^+^ autoreactive T cells found in NOD islets recognize the islet-specific glucose-6phosphatase catalytic subunit-related protein (IGRP), and play an important role in autoimmune disease recurrence after islet substitution (17). Using pentamer flow technology (representative gates for IGRP^+^CD8^+^ T cells in Supplementary Figure 5A), we quantified IGRP^+^CD8^+^ T cells in the KLN (Figure 3A) and islet grafts (Figure 3B). IGRP^+^CD8^+^ T cells were only rarely detectable 10 days after therapy initiation in the KLN of each group (Figure 3A). However, 6 weeks after therapy initiation, they were more prevalent at this site in the non-cured mice, irrespective of therapy (Figure 3A). When left untreated, IGRP^+^CD8^+^ T cells were highly present in islet grafts after 10 days, comprising on average 12.7% (±3.3%) of the entire CD8^+^ T cell population (Figure 3B), demonstrating that these cells may rapidly respond to islets without first clonally expanding in the KLN. Low-dose aCD3 therapy, with or without bacteria secreting PINS along with IL-10, significantly reduced the frequency to 1.3% (±0.7%) (Figure 3B). These reduced IGRP^+^CD8^+^ T cell frequencies persisted until 6 weeks after therapy initiation, though, mainly in mice cured by either therapy (Figure 3B).

**Figure 4:**
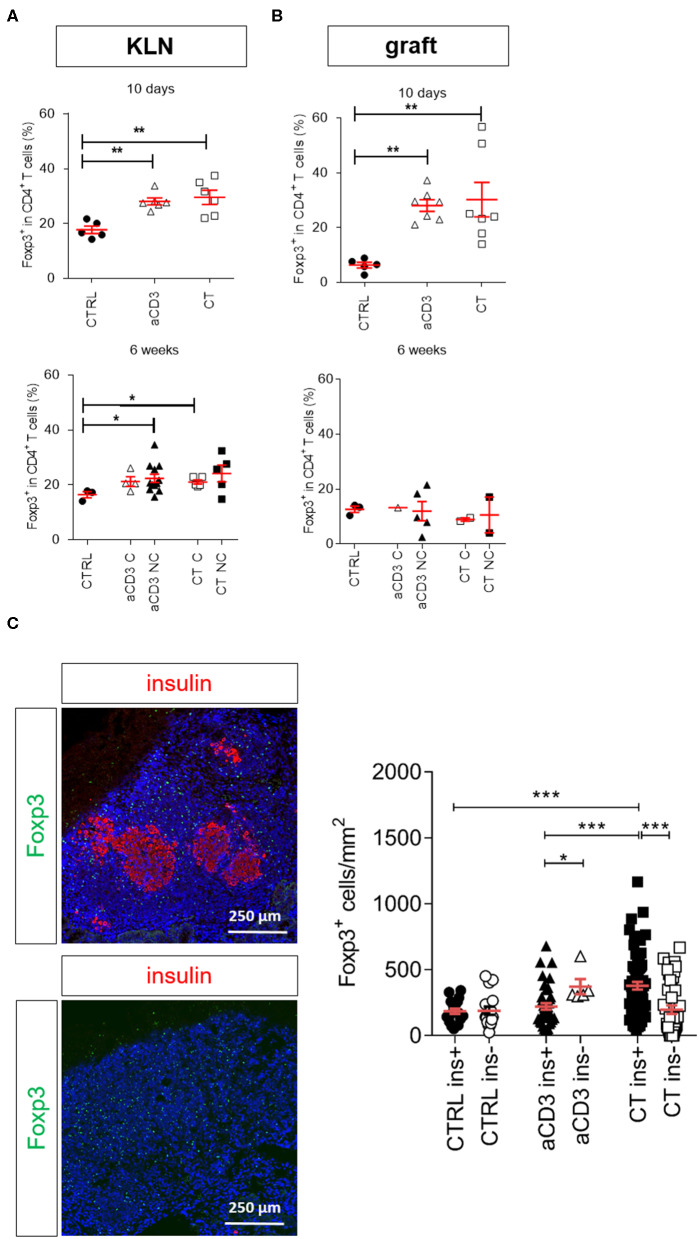
Combination treatment increases Foxp3^+^CD4^+^ Tregs in the islet grafts. Frequency of Foxp3^+^ cells within CD4^+^ T cell gate is shown at both 10 days and 6 weeks after islet substitution and therapy initiation (cured) or at disease recurrence (untreated and non-cured) in the kidney draining lymph nodes (KLN) **(A)** and islet grafts **(B)** of diabetic NOD mice. Symbols represent individual mice, and line and error bars reflect group mean ± SEM. Open symbols = cured [C], filled symbols = non-cured [NC]. **(C)** Representative immunofluorescence images showing Foxp3^+^ Tregs (green) surrounding the insulin (red)-positive islets (upper panel) and insulin (red)-negative islets (lower panel), grafted under the kidney capsule of a diabetic mouse cured by the combination therapy and retrieved 6 weeks after therapy initiation. DAPI was used as nuclear stain (blue). Photomicrograph is representative of 16 sections. The absolute numbers of Foxp3^+^ cells in the islet grafts was determined after 6 weeks of therapy initiation or at disease recurrence by manual counting on immunostained cryosections. Each symbol represents the Foxp3^+^ density per section, line and error bars reflect group mean ± SEM and equal sections were sampled per mouse (*n* = 1–4 mice per group). Black symbols indicate sections with insulin-positive islets (ins+). White symbols indicate sections with insulin-negative islets (ins-). Mice were left untreated (CTRL) or given a short-term low-dose aCD3 therapy (aCD3) either alone or combined with *L. lactis* bacteria secreting PINS with IL-10 (CT). Statistical significance between groups was calculated by Mann-Whitney *U* test; *P < 0.05, **P < 0.01, ***P < 0.001.

In previous settings, we demonstrated that low-dose aCD3 therapy and CT increased Foxp3^+^CD4^+^ Tregs in newly diagnosed diabetic mice (10, 11, 18). In the present longstanding diabetic model, Foxp3^+^ Treg frequencies were significantly higher in the KLN of both treatment groups compared to untreated controls after 10 days (Figure 4A). At this time, the majority of the Tregs expressed the IL-2Rα CD25, and the percentage of CD25^+^Foxp3^+^ Tregs was significantly increased (representative gates for CD25^+/−^Foxp3^+^ T cells in Supplementary Figures 5B, 6A). After 6 weeks or at disease recurrence, Treg frequencies were comparably elevated in the KLN of aCD3- and CT-treated mice compared to untreated controls (Figure 4A). In particular, the CD25^−^Foxp3^+^ Treg frequency was increased in the KLN of all treated mice, irrespective of therapeutic outcome (Supplementary Figure 6C). When analyzing the islet graft, both aCD3 therapy and CT similarly augmented the frequency of Foxp3^+^ Tregs, particularly the CD25^−^Foxp3^+^ subset, 10 days after therapy initiation (Figure 4B and Supplementary Figure 6B). At the graft site, these cells need to become activated and subsequently migrate to the KLN in order to suppress the autoimmune response and limit beta cell destruction. In fact, 6 weeks after therapy initiation or at disease recurrence, Foxp3^+^ Tregs seemed to have partially emigrated from the islet graft to the KLN (Figures 4A,B and Supplementary Figure 6D). Interestingly, the density of Foxp3^+^ Tregs was significantly higher in the islet grafts of CT-cured mice compared to values obtained in islet grafts from aCD3-cured mice (Supplementary Figure 7). Moreover, higher numbers of Foxp3^+^ cells were found around the insulin-containing islets in CT-treated mice compared to numbers counted in islet grafts of aCD3-treated or untreated controls (Figures 4B,C).

### Delivery of a Disease-Relevant Antigen by *L. lactis* Bacteria Combined With Low-Dose aCD3 Therapy Drives Insulin-Reactive Foxp3^+^CD4^+^ T Cells Into the Islet Graft

**Figure 5:**
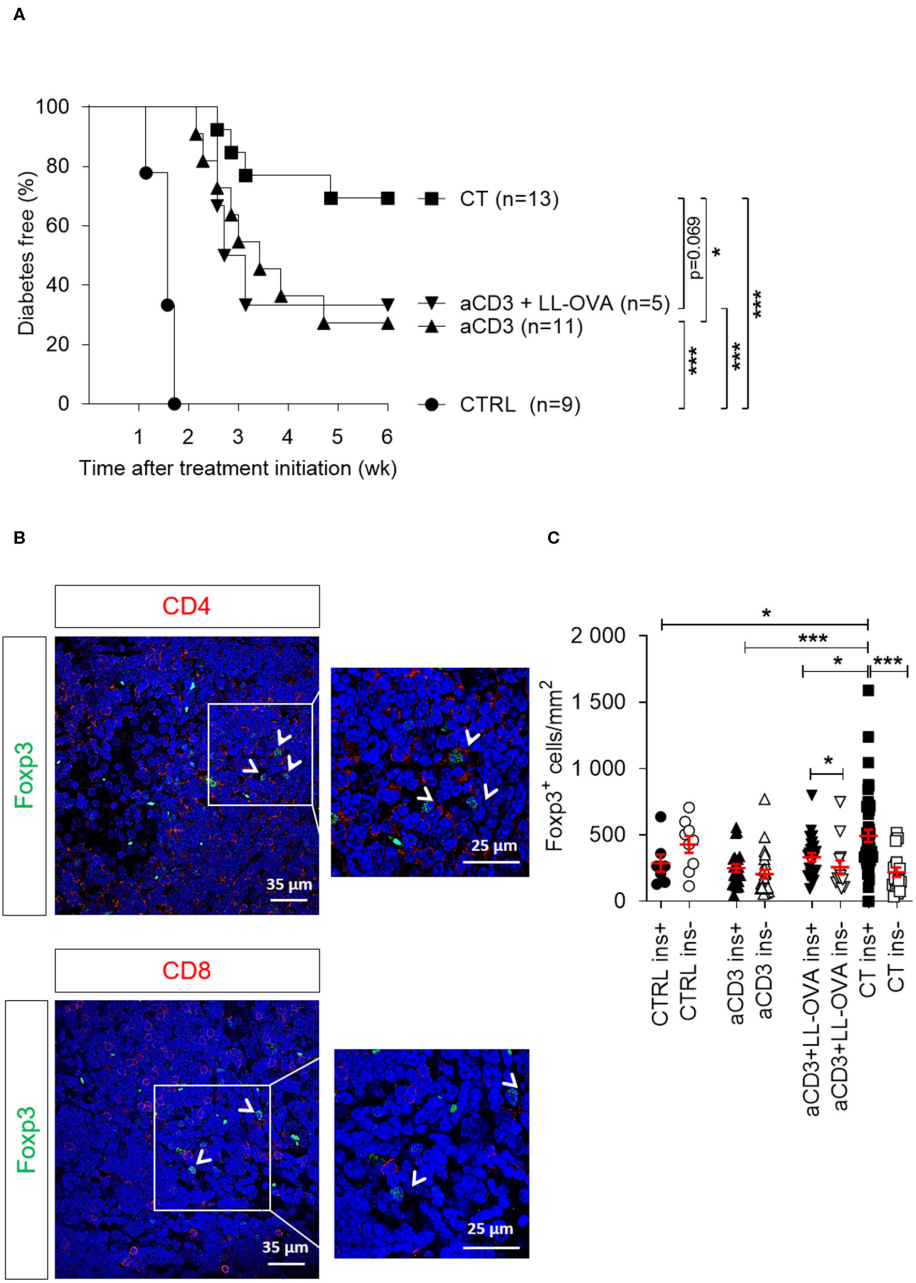
Delivery of a disease-relevant antigen by genetically modified *L. lactis* bacteria combined with low-dose aCD3 therapy drives Foxp3^+^CD4^+^ T cells into the islet grafts. Newly diagnosed diabetic NOD mice with disease duration of <2 days were injected i.v. with alloxan (90 mg/kg) and transplanted with 500 syngeneic islets after 48 h. Mice were left untreated (CTRL, *n* = 9) or given a short-term lowdose aCD3 therapy (aCD3; *n* = 11) either alone or combined with *L. lactis* bacteria secreting the irrelevant antigen ovalbumin (LL-OVA; *n* = 5) or secreting beta cell antigen (PINS, *n* = 13) combined with IL-10 (CT). **(A)** Shown is the percentage of mice remaining normoglycemic 6 weeks after therapy initiation as Kaplan-Meier survival curves. **(B)** Representative immunofluorescence images showing Foxp3^+^ Tregs in close contact with non-regulatory CD4^+^ (upper panel) and CD8^+^ (lower panel) T cells in islets grafted under the kidney capsule of a diabetic mouse cured by the combination therapy and retrieved 6 weeks after therapy initiation. DAPI was used as nuclear stain (blue). Photomicrographs are representative of 10 sections. Arrows depict Foxp3^+^ cells. **(C)** The absolute numbers of Foxp3^+^ cells in the islet grafts was determined 6 weeks after islet substitution and therapy initiation or at disease recurrence by manual counting on immunostained cryosections and displayed as mean ± SEM. Each symbol represents the Foxp3^+^ density per section, and equal sections were sampled per mouse (*n* = 2–4 mice per group). Black symbols indicate sections with insulin-positive islets (ins+). White symbols indicate sections with insulinnegative islets (ins-). Statistical significance between groups was calculated by log-rank **(A)** or MannWhitney *U* test **(C)**; *P < 0.05, **P < 0.01, ***P < 0.001.

To examine whether the type of antigen being orally delivered by *L. lactis* affects islet protection and Foxp3^+^ Treg migration and accumulation in target tissue, newly diagnosed diabetic mice were injected with alloxan to eliminate the endogenous beta cell pool (which may act as a source of local antigen), transplanted with syngeneic islets, and then treated either with CT containing the disease-relevant antigen PINS or an irrelevant antigen OVA, or treated with aCD3 therapy alone (see regimen in Supplementary Figure 1B). In this setting, the CT with *L. lactis* bacteria secreting PINS and IL-10 protected 69% of the NOD islet recipients (Figure 5A), in contrast to 33% when receiving an analogous CT therapy with OVA. Mice treated only with low-dose aCD3 therapy also had only 27% protection against disease recurrence, indicating that the presence of the islet antigen, in this case PINS, along with IL-10 seems to be crucial for Treg trafficking to the graft to induce peripheral immune tolerance to islet antigens. Foxp3^+^ Tregs, positive for CD4 yet not for CD8, were in close contact with non-regulatory CD4^+^ or CD8^+^ T cells (Figure 5B). The absolute numbers of Foxp3^+^ Tregs around the pancreatic islets still containing insulin was significantly enriched in mice receiving CT including PINS, compared to mice receiving CT including OVA or aCD3 therapy alone (Figure 5C).

**Figure 6:**
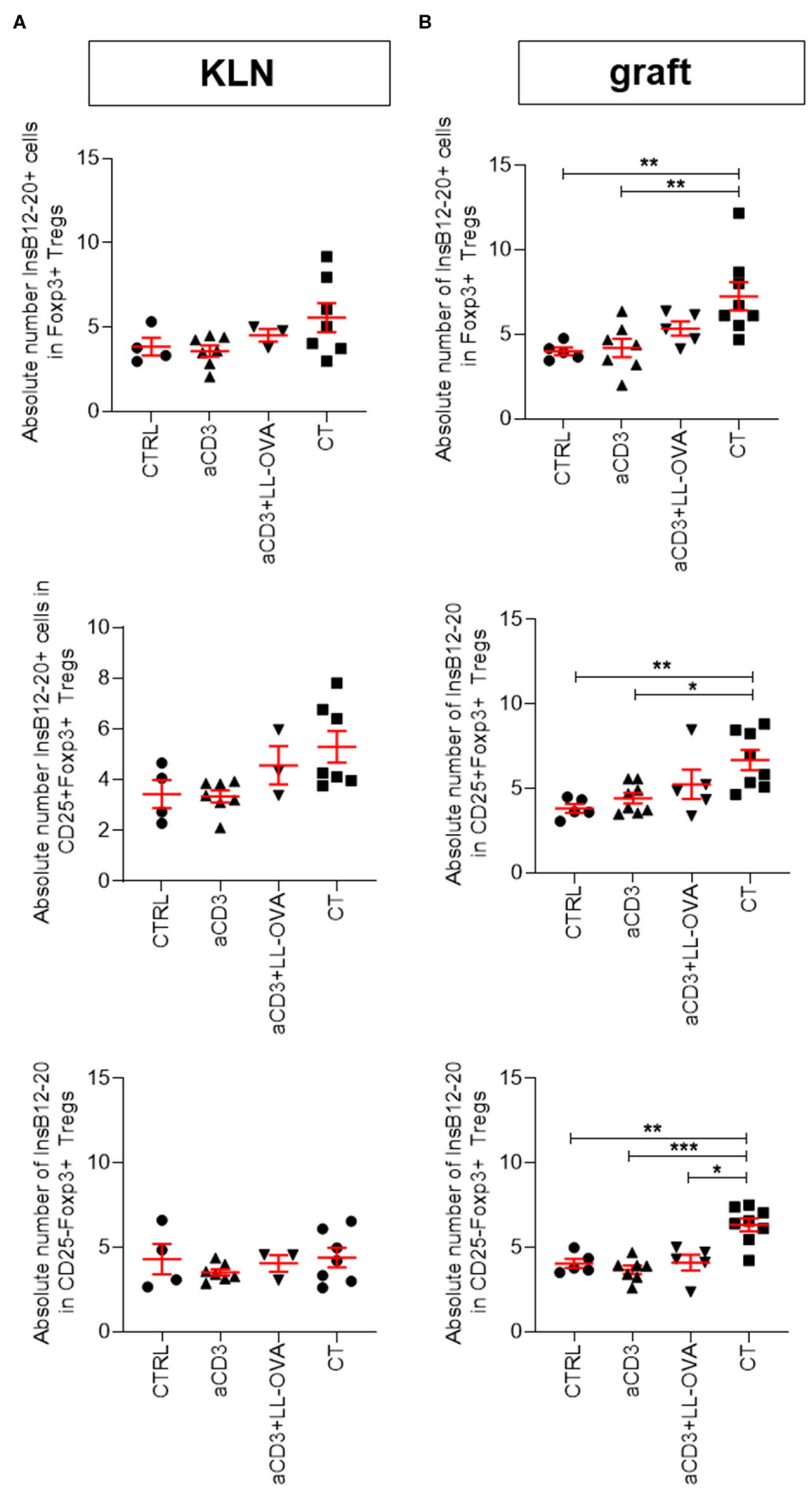
Combination therapy increases numbers of InsB12–20^+^Foxp3^+^CD4^+^ T cells in islet grafts. Newly diagnosed diabetic NOD mice with disease duration of <2 days were injected i.v. with alloxan (90 mg/kg) and transplanted with 500 syngeneic islets after 48 h. Mice were left untreated (CTRL; *n* = 4–5) or given a short-term low-dose aCD3 therapy (aCD3; *n* = 7–8) either alone or combined with *L. lactis* bacteria secreting the irrelevant antigen ovalbumin (LL-OVA; *n* = 3–5) or secreting beta cell antigen (PINS; *n* = 7–8) combined with IL-10 (CT). Absolute numbers of tetramer positive (tet^+^) InsB12–20 cells per 100 Foxp3^+^ Tregs, either CD25^+^ or CD25^−^,are shown 3 weeks after islet substitution and therapy initiation in the kidney draining lymph nodes (KLN) **(A)** and islet grafts **(B)** of diabetic NOD mice. Symbols represent individual mice, and line and error bars reflect group mean ± SEM. Statistical significance between groups was calculated by Mann-Whitney *U* test; *P < 0.05, **P < 0.01, ***P < 0.001.

Having access to new MHC class II tetramers in combination with CountBright™ absolute counting beads allowed us to detect absolute number of Foxp3^+^ Tregs reactive to two adjacent registers in the B9–23 segment of insulin (15). Interestingly, increased numbers of Foxp3^+^ Tregs, both CD25^+^ and CD25^−^, detected in the islet grafts, but not in the KLN, of the combination therapy-treated mice were reactive to InsB12–20 and to lesser extent to InsB13–21 (Figures 6A,B and Supplementary Figures 8A,B). Only in the mice treated with the add-on of the islet antigen proinsulin, a higher number of insulin-reactive Tregs in the islet grafts was observed compared to mice treated with anti-CD3 (with or without OVA) therapy alone or the untreated controls (Figure 6B and Supplementary Figure 8B).

## Discussion

In the present study, we investigated the potential for oral delivery of human PINS and IL-10 by genetically modified *L. lactis*, combined with a short low-dose aCD3 therapy, to reestablish tolerance to (engrafted) beta cells in longstanding diabetic NOD mice. The use of aCD3 as a monotherapy was already successful in inducing disease remission in newly diagnosed diabetic mice (9, 19) and when combined with the tolerogenic delivery of islet antigens (PINS or a GAD65 fragment via *L. lactis*), reached approximately 60% disease remission (10, 18). A clinical trial is currently underway to assess the safety and tolerability of AG019, *L. lactis* delivering human PINS and IL-10, administered alone or in combination with Teplizumab in newly diagnosed T1D patients (ClinicalTrials.gov Identifier: NCT03751007).

We postulate that mice not curing by CT may have had insufficient beta cell mass at start to reach metabolic control, especially since mice with mild starting blood glucose concentrations (<350 mg/dL) had a superior disease remission rate (10, 11). Besides, non-cured mice did respond immunologically to CT, as demonstrated by the strong accumulation of Foxp3^+^ Tregs in the pancreas (11). Until now, the potential of aCD3 has not been intensively investigated outside the narrow window of disease diagnosis. Here, we transplanted 500 insulitis-free syngeneic NOD islets into long-duration diabetic mice to restore metabolic control and investigate the potential of the antigen-based CT to establish tolerance even after variable disease durations.

We previously showed in a small cohort of mice that low-dose aCD3 strongly delayed disease recurrence after islet substitution (14). Here, we followed a larger cohort of mice and saw 32% of aCD3-treated mice maintaining normoglycemia and when combined with *L. lactis* bacteria secreting PINS with IL-10 the success rate reached 48%. An important observation is that longer disease duration, specifically to the point of lacking measurable beta cell function, before therapy initiation diminished therapeutic success. However, CT-treated mice were significantly better at avoiding disease recurrence, and therapeutic success was less influenced by disease duration. We propose this is, in part, related to the preservation of residual pancreatic beta cell mass at therapy initiation, since we previously established that CT does not stimulate pancreatic beta cell regeneration (10). Many T1D patients are shown to maintain some functional beta cell mass after diagnosis, indicated by detectable C-peptide or insulin RNA/PINS protein, some even after disease duration of over 30 years (5, 7). These people may benefit more from tolerance-restoring interventions that preserve or revive remaining beta cells.

The mechanisms by which aCD3 monotherapy and CT exert benefit may be dual. These regimens may alleviate (recurrent) autoimmunity directed at (engrafted) beta cells by temporarily eliminating the autoreactive T cells, and by promoting the generation of Tregs, and in case of CT, islet antigen-specific Tregs. The data here point to the necessity of aCD3 in CT to prevent early islet infiltration by autoreactive IGRP^+^CD8^+^ T cells that are involved in beta cell destruction in both mice and humans (17). Antigen-experienced IGRP^+^CD8^+^ T cells rapidly accumulate in newly implanted islets, expressing antigen locally, expand while acquiring an effector-memory phenotype and then traffic to the draining LN, the site of activation of naive autoreactive CD8^+^ T cells (20). Although we did not study the activation/memory status of IGRP^+^CD8^+^ T cells, the present data revealed that therapy-cured mice continued to have low frequencies of IGRP^+^CD8^+^ T cells after 6 weeks, whereas therapy non-cured mice showed frequencies similar to untreated controls, suggestive of a higher degree of clonal expansion in the islets, particularly in the KLN. Studies on autoreactive effector T cells have shown that Tregs can prevent clonal expansion by limiting access to antigen-presenting cells (21). Here, we found that mice treated with aCD3 alone or combined with *L. lactis* bacteria secreting PINS with IL-10 had similar frequencies of Foxp3^+^ Tregs present in the graft and KLN shortly after therapy initiation. However, 6 weeks after therapy initiation, Foxp3^+^ Tregs seemed to have partially emigrated from the islet grafts to the KLN. Some interesting studies demonstrated that the presence of Tregs in islets is insufficient to prevent infiltration of autoreactive T cells, suggesting that migration of Tregs into to draining LN may be necessary to sustain tolerance (22, 23). Of note, the absolute number of Tregs closely surrounding insulin-containing islets was increased only by the CT, eluding to their potential antigen specificity.

We previously demonstrated that CT established long-term tolerance to islet antigens in newly diagnosed diabetic mice by generating antigen-specific Foxp3^+^CTLA4^+^ Tregs with superior regulatory properties when challenged with the respective autoantigen, without eliminating the autoreactive T cell compartment, implying a resetting of the immunological balance (10, 11). Using a humanized mouse, Waldron-Lynch et al. reported that aCD3 induced only a modest and transient T cell depletion, but alternatively triggered the generation of a “gut-homing” CD4^+^CD25^hi^CCR6^+^Foxp3^+^ Treg population, which produced IL-10 on migration to the gut and subsequently returned to the circulation (24). Nishio et al. further demonstrated that Treg expansion by aCD3 occurred not through conversion from Foxp3^−^ conventional T cells, but via expansion of a monoclonal Treg population, normally selected and sustained at low frequency (25). Interestingly, they also proposed that a minor cytokine storm elicited by aCD3 may actually be a crucial contributing element in the therapeutic effect, via resetting of the Treg niches. We suggest that, when combined with an islet antigen delivered via the tolerogenic (oral) route, low-dose aCD3 may encourage specific modifications in the homeostatic control of the islet antigen-specific repertoire or a phenotypic redistribution, rather than a general Treg expansion.

The present data support the idea that CT is superior due to the tolerogenic delivery of a diseaserelevant antigen (i.e., PINS) which may preferentially generate or expand islet insulin-reactive Tregs being directed to the intestine by CD3 antibodies which are known to be more suitable for controlling autoimmunity *in situ* than polyclonal Tregs, especially when the islet antigen is still on board (26, 27). By eliminating the local antigen source before therapy initiation using alloxan, we showed the importance of continuous islet antigen feeding on therapeutic success of CT. As we see less Foxp3^+^ Tregs around the transplanted insulin-containing islets of aCD3-treated mice (regardless of the presence of OVA), compared to CT-treated mice, autoreactive T cells may be less restrained and can cause further islet destruction due to epitope spreading which recruits other pathogenic effectors. Interestingly, when the combination therapy with the islet antigen PINS was used, increased numbers of insulin-reactive Foxp3^+^ Tregs were observed in the islet grafts, yet not in the KLN, indicating that these cells had trafficked to the inflammatory sites where they may suppress persistent effector T cell function. Moreover, the present data suggest that Tregs may control disease recurrence by first migrating to the islet grafts to limit beta cell damage (day 10 post therapy initiation), and then partly retreating to the KLN to maintain tolerance (6 weeks post therapy initiation).

Taken together, CT greatly reduced IGRP^+^CD8^+^ graft infiltration and increased insulin-reactive Foxp3^+^ Treg accumulation, allowing Tregs to dominate around insulin-containing islets. We speculate that these Tregs may persistently interact with memory (autoantigen-experienced) autoreactive T cells, but an imbalance in their numbers might determine the outcome. More insights are needed on how these Tregs control the diverse repertoire of antigen-experienced T cells present in the islets (28). Tregs can create a tolerogenic milieu that promotes bystander suppression and infectious tolerance through several mechanisms (29).

In conclusion, low-dose aCD3 therapy in combination with oral delivery of *L. lactis* bacteria secreting PINS with IL-10 can bypass autoimmune recurrence in syngeneic islet recipients with long-duration T1D. Therapeutic success depended on disease duration and, thus, in part on endogenous beta cell mass, but also on the continuous oral delivery of islet antigen. Islet (insulin-reactive) Tregs were enriched and curbed progression to beta cell destruction. Integration of these antigen-specific approaches to enhance tolerance and control autoimmunity may help advance therapeutic efficacy in selected patient populations even beyond the window of disease diagnosis.

## Data Availability Statement

The datasets generated for this study are available on request to the corresponding author.

## Ethics Statement

The animal study was reviewed and approved by KULeuven Animal Care and Use Committee.

## Author Contributions

DC, JC, and CG designed the study and performed experiments, interpreted data, and drafted the manuscript. P-JM, GSa, FM, GV, GSe, A-SV, MA, KV, LS, SC, PR, LT, FD, and CM provided substantial contributions to conception and design, acquisition of data, analysis and interpretation, as well as critically revising the article. CG and CM stand as the guarantors of this work and, as such, had full access to all the data in the study and take responsibility for the integrity of the data and the accuracy of the data analysis. All authors gave final approval of the version to be published.

## Conflict of Interest

KV, SC, LS, and PR have financial interests in ActoBio Therapeutics™, including employment and stock options. The remaining authors declare that the research was conducted in the absence of any commercial or financial relationships that could be construed as a potential conflict of interest.

## Abbreviations

aCD3: anti-CD3
ATG: anti-thymocyte globulin
CFU: colony forming unit
CT: combination treatment
FcR: Fc receptor
IGRP: islet-specific glucose-6-phosphatase catalytic subunit-related protein
KLN: kidney draining lymph nodes
L. lactis: Lactococcus lactis
OVA: ovalbumin
PINS: proinsulin
T1D: type 1 diabetes
Tcm: central memory T cell
Tem: effector memory T cell
Tn: naïve T cell
Treg: regulatory T cell.

## References

[1] Haller MJ Schatz DA Skyler JS Krischer JP Bundy BN Miller JL. Type 1 diabetes trialnet, Low-dose anti-thymocyte globulin (ATG) preserves beta-cell function and improves HbA1c in newonset type 1 diabetes. Diabetes Care. (2018) 41:1917–25. 10.2337/dc18-0494 30012675 PMC6105329

[2] Orban T Monzavi R Moran A Peakman M Raskin P Russell WE. Type 1 diabetes trialnet abatacept study, costimulation modulation with abatacept in patients with recent-onset type 1 diabetes: follow-up 1 year after cessation of treatment. Diabetes Care. (2014) 37:1069–75. 10.2337/dc13-0604 24296850 PMC3964491

[3] Keymeulen B Vandemeulebroucke E Ziegler AG Mathieu C Kaufman L Hale G. Insulin needs after CD3-antibody therapy in new-onset type 1 diabetes. N Engl J Med. (2005) 352:2598–608. 10.1056/NEJMoa043980 15972866

[4] Sherry NA Tsai EB Herold KC. Natural history of beta-cell function in type 1 diabetes. Diabetes. (2005) 54(Suppl. 2):S32–9. 10.2337/diabetes.54.suppl_2.S32 16306337

[5] Wang L Lovejoy NF Faustman DL. Persistence of prolonged C-peptide production in type 1 diabetes as measured with an ultrasensitive C-peptide assay. Diabetes Care. (2012) 35:465–70. 10.2337/dc11-1236 22355018 PMC3322715

[6] Oram RA Jones AG Besser RE Knight BA Shields BM Brown RJ. The majority of patients with long-duration type 1 diabetes are insulin microsecretors and have functioning beta cells. Diabetologia. (2014) 57:187–91. 10.1007/s00125-013-3067-x 24121625 PMC3855529

[7] Wasserfall C Nick HS Campbell-Thompson M Beachy D Haataja L Kusmartseva I. Persistence of pancreatic insulin mRNA expression and proinsulin protein in type 1 diabetes pancreata. Cell Metab. (2017) 26:568–75. 10.1016/j.cmet.2017.08.013 28877460 PMC5679224

[8] Van Belle TL Juntti T Liao J von Herrath MG. Pre-existing autoimmunity determines type 1 diabetes outcome after Flt3-ligand treatment. J Autoimmun. (2010) 34:445–52. 10.1016/j.jaut.2009.11.010 20004555 PMC2860005

[9] Chatenoud L Primo J Bach JF. CD3 antibody-induced dominant self tolerance in overtly diabetic NOD mice. J Immunol. (1997) 158:2947–54. 9058834

[10] Takiishi T Korf H Van Belle TL Robert S Grieco FA Caluwaerts S. Reversal of autoimmune diabetes by restoration of antigen-specific tolerance using genetically modified *Lactococcus lactis* in mice. J Clin Invest. (2012) 122:1717–25. 10.1172/JCI60530 22484814 PMC3336982

[11] Takiishi T Cook DP Korf H Sebastiani G Mancarella F Cunha JP. Reversal of diabetes in NOD mice by clinical-grade proinsulin and IL-10-secreting *Lactococcus lactis* in combination with lowdose anti-CD3 depends on the induction of Foxp3-positive T cells. Diabetes. (2017) 66:448–59. 10.2337/db15-1625 28108611

[12] Unger WW Mulder A Franken KL Hilbrands R Keymeulen B Peakman M. Discovery of low-affinity preproinsulin epitopes and detection of autoreactive CD8 T-cells using combinatorial MHC multimers. J Autoimmun. (2011) 37:151–9. 10.1016/j.jaut.2011.05.012 21636247

[13] Huibregtse IL Snoeck V de Creus A Braat H De Jong EC Van Deventer SJ. Induction of ovalbumin-specific tolerance by oral administration of *Lactococcus lactis* secreting ovalbumin. Gastroenterology. (2007) 133:517–28. 10.1053/j.gastro.2007.04.073 17681173

[14] Baeke F Van Belle TL Takiishi T Ding L Korf H Laureys J. Low doses of anti-CD3, ciclosporin A and the vitamin D analogue, TX527, synergise to delay recurrence of autoimmune diabetes in an islet-transplanted NOD mouse model of diabetes. Diabetologia. (2012) 55:2723–32. 10.1007/s00125-012-2630-1 22752077

[15] Gioia L Holt M Costanzo A Sharma S Abe B Kain L. Position beta57 of I-A^g7^ controls early anti-insulin responses in NOD mice, linking an MHC susceptibility allele to type 1 diabetes onset. Sci Immunol. (2019) 4. 10.1126/sciimmunol.aaw6329 31471352 PMC6816460

[16] Weiner HL. Oral tolerance, an active immunologic process mediated by multiple mechanisms. J Clin Invest. (2000) 106:935–7. 10.1172/JCI11348 11032852 PMC314352

[17] Wong CP Li L Frelinger JA Tisch R. Early autoimmune destruction of islet grafts is associated with a restricted repertoire of IGRP-specific CD8^+^ T cells in diabetic nonobese diabetic mice. J Immunol. (2006) 176:1637–44. 10.4049/jimmunol.176.3.1637 16424193

[18] Robert S Demetter P Wasserfall CH Atkinson MA Dotta F Rottiers P. Oral delivery of glutamic acid decarboxylase (GAD)-65 and IL-10 by *Lactococcus lactis* reverses diabetes in recent-onset NOD mice. Diabetes. (2014) 63:2876–87. 10.2337/db13-1236 24677716

[19] Kohm AP Williams JS Bickford AL McMahon JS Chatenoud L Bach JF. Treatment with nonmitogenic anti-CD3 monoclonal antibody induces CD4+ T cell unresponsiveness and functional reversal of established experimental autoimmune encephalomyelitis. J Immunol. (2005) 174:4525–34. 10.4049/jimmunol.174.8.4525 15814673

[20] Alkemade GM Clemente-Casares X Yu Z Xu BY Wang J Tsai S. Local autoantigen expression as essential gatekeeper of memory T-cell recruitment to islet grafts in diabetic hosts. Diabetes. (2013) 62:905–11. 10.2337/db12-0600 23160528 PMC3581210

[21] Tang Q Adams JY Tooley AJ Bi M Fife BT Serra P. Visualizing regulatory T-cell control of autoimmune responses in nonobese diabetic mice. Nat Immunol. (2006) 7:83–92. 10.1038/ni1289 16311599 PMC3057888

[22] Sarween N Chodos A Raykundalia C Khan M Abbas AK Walker LS. CD4^+^CD25^+^ cells controlling a pathogenic CD4 response inhibit cytokine differentiation, CXCR3 expression, and tissue invasion. J Immunol. (2004) 173:2942–51. 10.4049/jimmunol.173.5.2942 15322152

[23] Zhang N Schroppel B Lal G Jakubzick C Mao X Chen D. Regulatory T cells sequentially migrate from inflamed tissues to draining lymph nodes to suppress the alloimmune response. Immunity. (2009) 30:458–69. 10.1016/j.immuni.2008.12.022 19303390 PMC2737741

[24] Waldron-Lynch F Henegariu O Deng S Preston-Hurlburt P Tooley J Flavell R. Teplizumab induces human gut-tropic regulatory cells in humanized mice and patients. Sci Transl Med. (2012) 4:118ra12. 10.1126/scitranslmed.3003401 22277969 PMC4131554

[25] Nishio J Feuerer M Wong J Mathis D Benoist C. Anti-CD3 therapy permits regulatory T cells to surmount T cell receptor-specified peripheral niche constraints. J Exp Med. (2010) 207:187989. 10.1084/jem.20100205 20679403 PMC2931163

[26] Tonkin DR He J Barbour G Haskins K. Regulatory T cells prevent transfer of type 1 diabetes in NOD mice only when their antigen is present *in vivo*. J Immunol. (2008) 181:4516–22. 10.4049/jimmunol.181.7.4516 18802054

[27] Sagoo P Ali N Garg G Nestle FO Lechler RI Lombardi G. Human regulatory T cells with alloantigen specificity are more potent inhibitors of alloimmune skin graft damage than polyclonal regulatory T cells. Sci Transl Med. (2011) 3:83ra42. 10.1126/scitranslmed.3002076 21593402 PMC3776382

[28] Toivonen R Arstila TP Hanninen A. Islet-associated T-cell receptor-beta CDR sequence repertoire in prediabetic NOD mice reveals antigen-driven T-cell expansion and shared usage of VbetaJbeta TCR chains. Mol Immunol. (2015) 64:127–35. 10.1016/j.molimm.2014.11.009 25480393

[29] Waldmann H. Tolerance can be infectious. Nat Immunol. (2008) 9:1001–3. 10.1038/ni0908-1001 18711438

