# Supplementary material for: Intestinal Delivery of Proinsulin and IL-10 via Lactococcus lactis Combined With Low-Dose Anti-CD3 Restores Tolerance Outside the Window of Acute Type 1 Diabetes Diagnosis
Source: Front Immunol. 2020 Jun 9;11:1103. doi: 10.3389/fimmu.2020.01103 (PMC7295939; doi:10.3389/fimmu.2020.01103)
Supplement: Supplementary file 1 [file Data_Sheet_1.PDF]

Supplementary Fig. 1

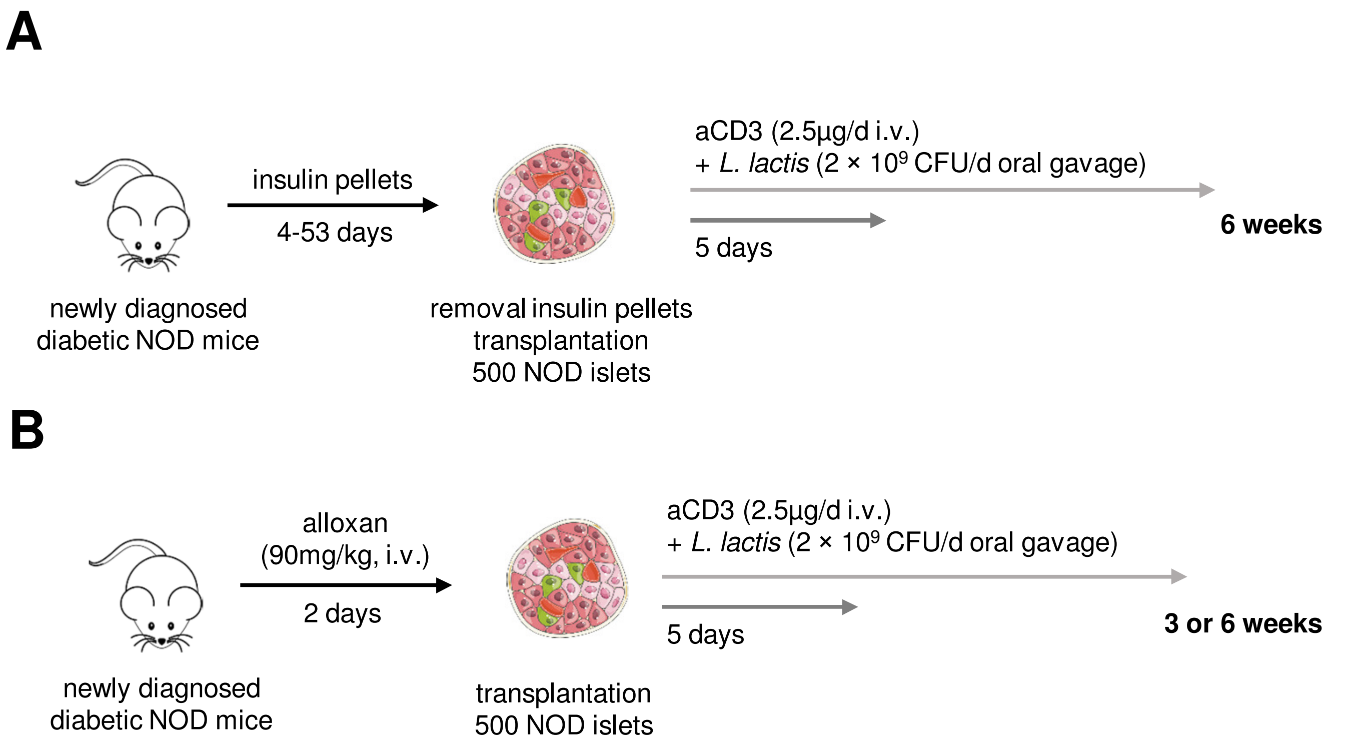

Supplementary Figure 1. Therapy regimen in long-duration T1D mice after islet substitution. Diabetic NOD mice with disease duration between 4 and 53 days were maintained on insulin pellets. At that stage, all mice received a syngeneic transplantation of 500 insulitis-free islets and were left untreated (**CTRL**) or given a short-term low-dose aCD3 therapy (**aCD3**; 2.5 µg/d i.v. for 5 consecutive days) either alone or combined with *L. lactis* bacteria secreting proinsulin and IL-10 (LL-PINS+IL-10; **CT**; 2 × 10^9 CFU/d during 6 weeks)(**A**). In a separate cohort, newly diagnosed diabetic NOD mice were injected intravenously with alloxan (90 mg/kg i.v.) in order to completely deplete residual beta cell mass. After 48 hours, all mice received 500 syngeneic islets and were left untreated (**CTRL**) or given a short-term low-dose aCD3 therapy (**aCD3**; 2.5 µg/d i.v. for 5 consecutive days) either alone or combined with *L. lactis* bacteria secreting ovalbumin + IL-10 (**aCD3+LL-OVA**; 2 × 10^9 CFU/d) or secreting proinsulin and IL-10 (LL-PINS+IL-10; **CT**; 2 × 10^9 CFU/d) for a period of 3 or 6 weeks (**B**).

Supplementary Fig. 2

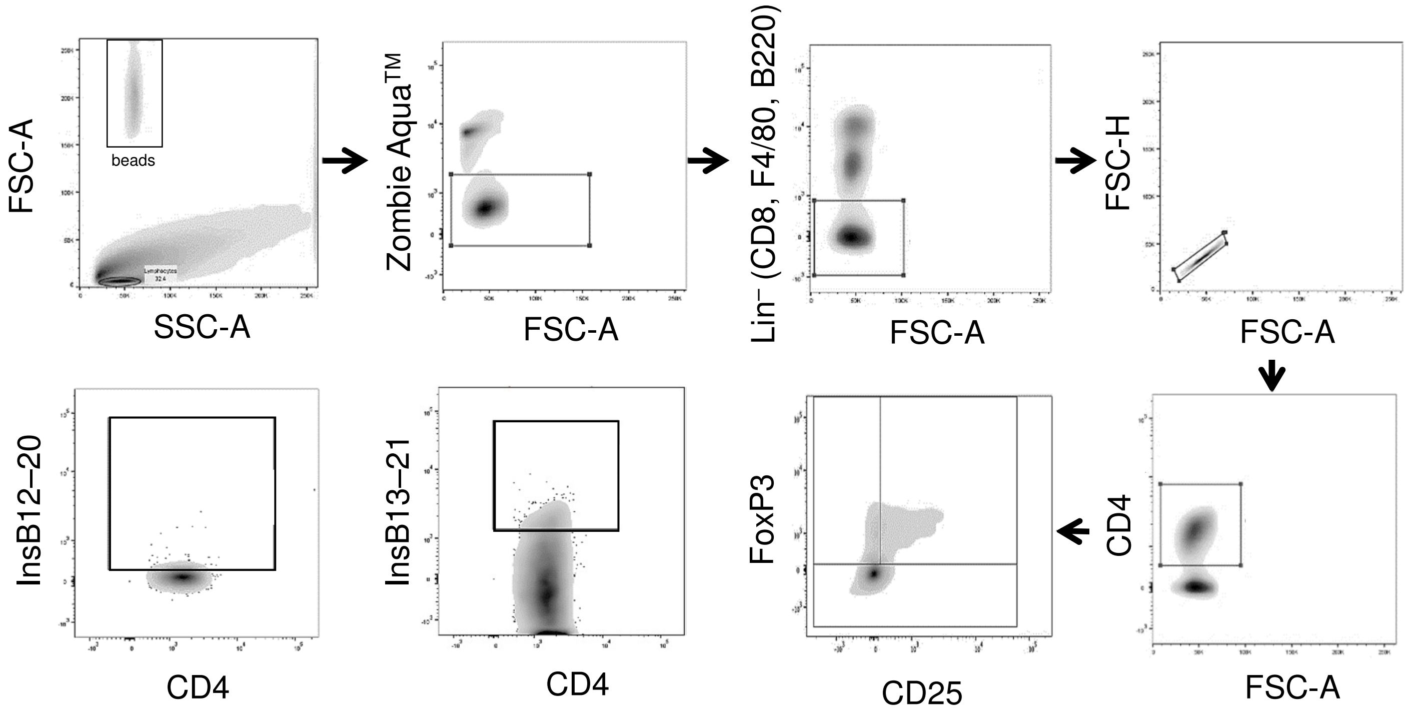

Supplementary Figure 2. Insulin-reactive (e.g., InsB12–20 and InsB13–21) Foxp3⁺ regulatory T cells were detected using PE- and APC-labeled MHC/peptide tetramers. Gates were set on FSC^{int} SSC^{int} (lymphocytes), live (Zombie Aqua™), Lin^{-} (CD8, F4/80, B220), single cells (FSC-A/FSC-H), $CD4^{+}$, $CD25^{+}$ and $Foxp3^{+}$. Values indicate the absolute number of tetramer positive (tet^{+}) InsB12–20 or InsB13–21 cells per 100 $Foxp3^{+}$ Tregs, either $CD25^{+}$ or $CD25^{-}$. Absolute numbers were obtained by including CountBright™ absolute counting beads.

Supplementary Fig. 3

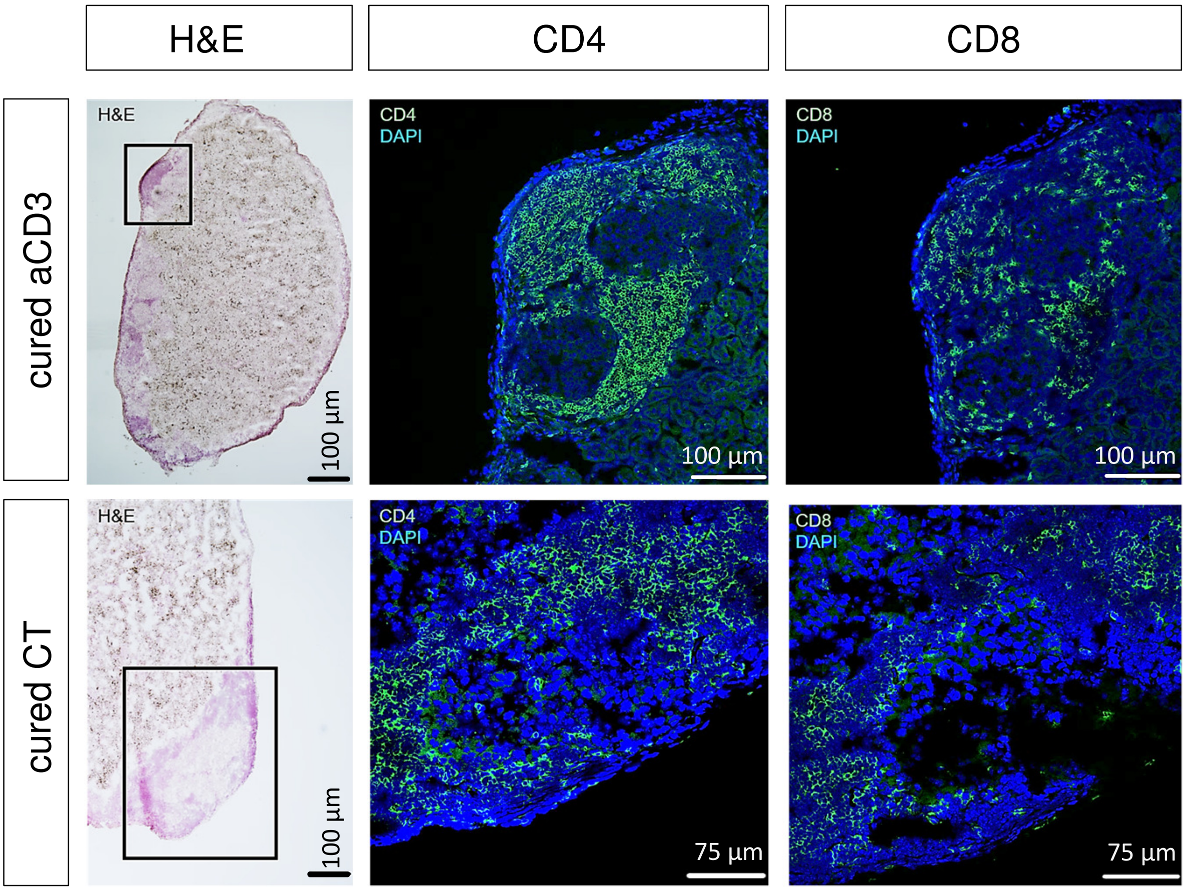

Supplementary Figure 3. Combination treatment endorses islet protection despite massive immune infiltration. Sections of islet grafts from cured mice of the indicated treatment groups (**aCD3**, and **CT**, combination treatment) were stained with hematoxylin and eosin (H&E). Adjacent sections were immunostained for CD4 and CD8. Grafts from therapy cured mice showed well-granulated islets, however, with accumulation of $CD4^{+}$ and $CD8^{+}$ T cells around the islets. Photomicrographs (scale bar 75 or 100 µm) are representative of 10 sections per recipient. DAPI was used as nuclear stain (blue). Staining patterns were consistent across animals studied.

Supplementary Fig. 4

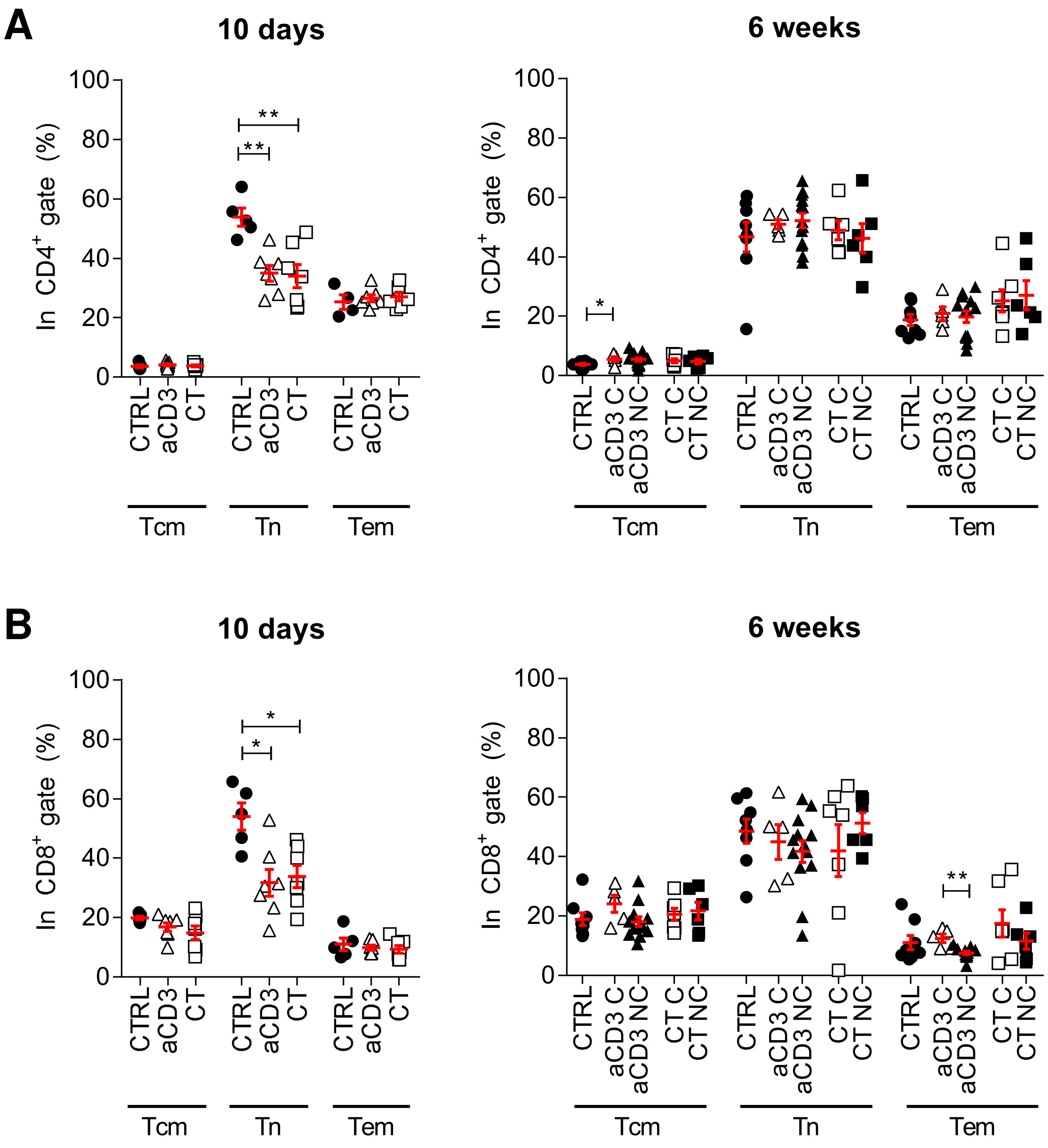

Supplementary Fig. 4

Supplementary Figure 4. Circulating naïve, effector and memory T cell frequencies are unaltered after combination treatment. Frequencies of naive (Tn, $CD44^{lo}CD62L^{+}$), effector memory (Tem, $CD44^{hi}CD62L^{-}$), and central memory (Tcm, $CD44^{hi}CD62L^{+}$) cells within the $CD4^{+}$ (**A**) and $CD8^{+}$ (**B**) T cell gate are shown as mean ± SEM in the spleen of diabetic NOD mice, being islet substituted more than 2 weeks after onset, at both 10 days and 6 weeks after therapy initiation (cured) or at disease recurrence (untreated and non-cured). Mice were left untreated (**CTRL**) or given a short-term low-dose aCD3 therapy (**aCD3**) either alone or combined with LL-PINS+IL-10 (**CT**). Symbols represent individual mice, and line and error bars reflect group mean ± SEM. Open symbols = cured [C], filled symbols = non-cured [NC]. Statistical significance between groups was calculated by Mann-Whitney U test; $*P < 0.05$, $**P < 0.01$.

Supplementary Fig. 5

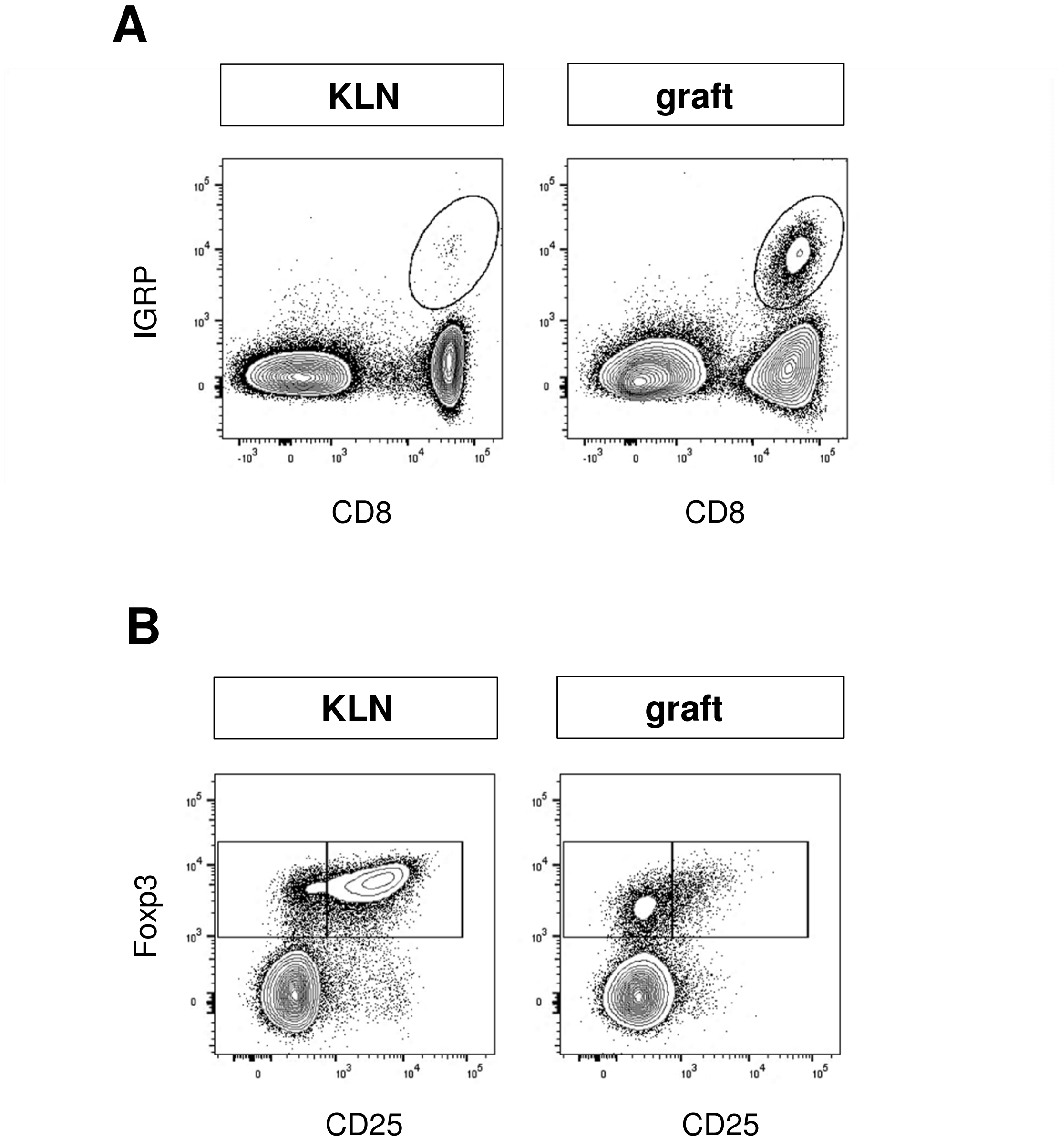

Supplementary Figure 5. Representative gating strategies for IGRP$^+$CD8$^+$ autoreactive T cells and CD25^{+}/$^{-}$Foxp3$^+$CD4$^+$ regulatory T cells. Representative flow cytometry plots showing the frequency of IGRP$^+$CD8$^+$ T cells using pentamer technology (**A**) and Foxp3 and CD25 expression within the CD4$^+$ T cell gate (**B**) in kidney draining lymph nodes (KLN) and islet grafts of an untreated NOD islet recipient 6 weeks after therapy initiation.

.

Supplementary Fig. 6

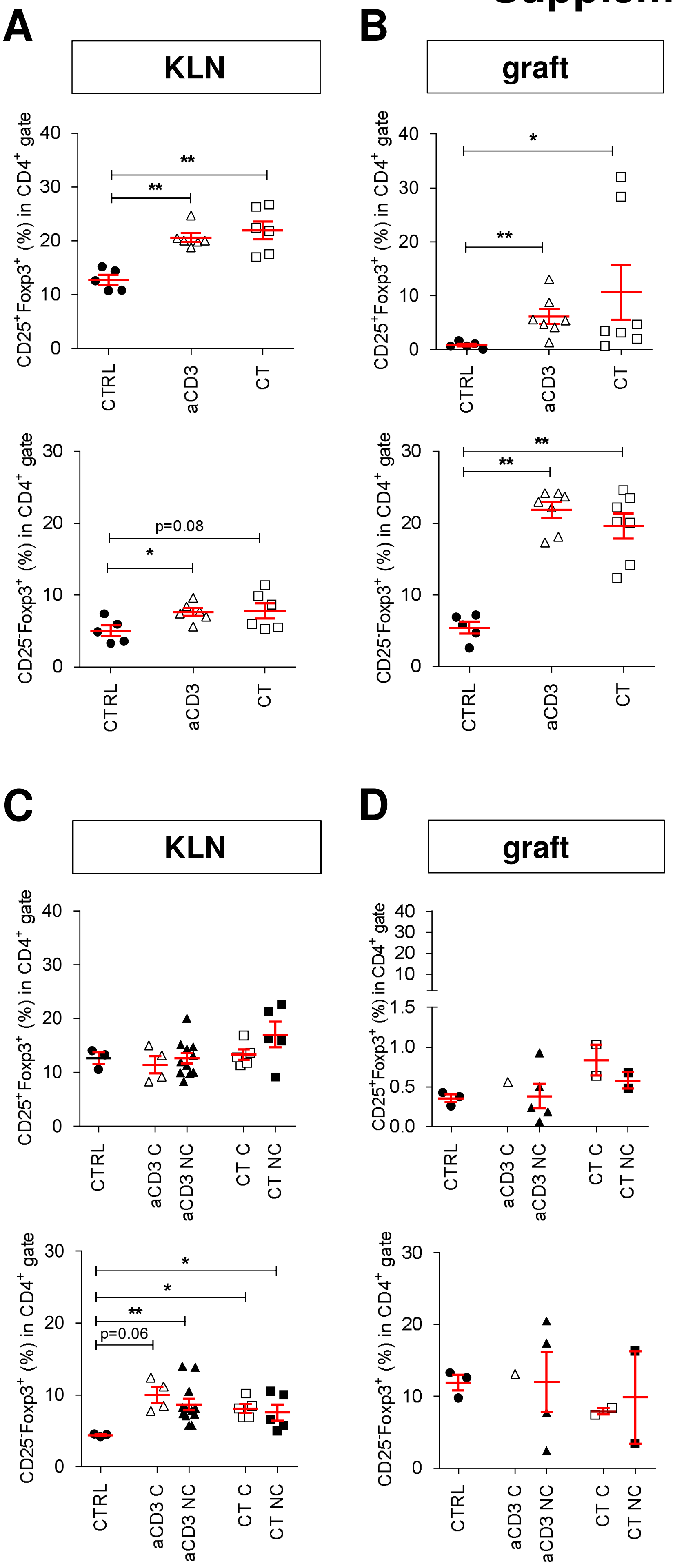

Supplementary Fig. 6

Supplementary Figure 6. Therapy-induced Tregs are enriched in CD25^{-}Foxp3^{+} T cells. Frequency of Foxp3^{+} cells within CD4^{+} T cell gate is shown at both 10 days (**A, B**) and 6 weeks (**C, D**) after islet substitution and therapy initiation (cured) or at disease recurrence (untreated and non-cured) in kidney draining lymph nodes (KLN) (**A, C**) and islet grafts (**B, D**). Mice were left untreated (**CTRL**) or given a short-term low-dose aCD3 therapy (**aCD3**) either alone or combined with LL-PINS+IL-10 (**CT**). Symbols represent individual mice, and line and error bars reflect group mean ± SEM. Open symbols = cured [C], filled symbols = non-cured [NC]. Statistical significance between groups was calculated by Mann-Whitney U test; *P < 0.05, **P < 0.01.

Supplementary Fig. 7

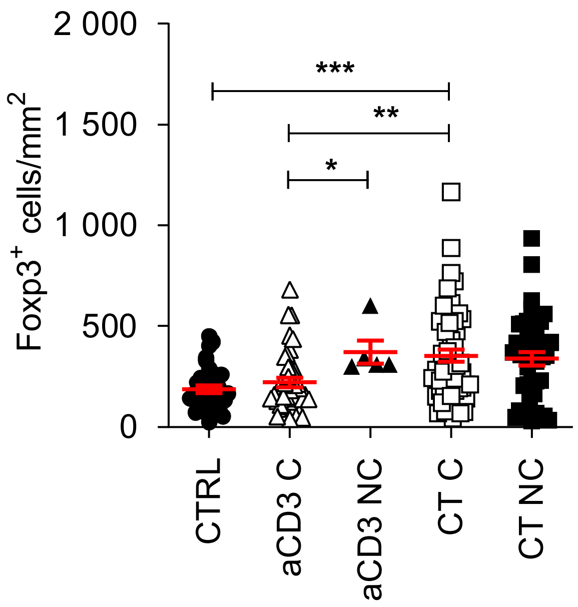

Supplementary Figure 7. Combination treatment increases Foxp3+ Tregs in the islet grafts. The absolute numbers of Foxp3+ cells in the islet graft was determined after 6 weeks of therapy initiation or at disease recurrence by manual counting on immunostained cryosections and displayed as mean ± SEM. Each symbol represents the Foxp3+ density per section, line and error bars reflect group mean ± SEM, and equal sections were sampled per mouse (n = 1–4 mice per group). Mice were left untreated (**CTRL**) or given a short-term low-dose aCD3 therapy (**aCD3**) either alone or combined with *L. lactis* bacteria secreting PINS with IL-10 (**CT**). Open symbols = cured [C], filled symbols = non-cured [NC]. Statistical significance between groups was calculated by Mann-Whitney U test; $*P < 0.05$, $**P < 0.01$, $***P < 0.001$.

Supplementary Fig. 8

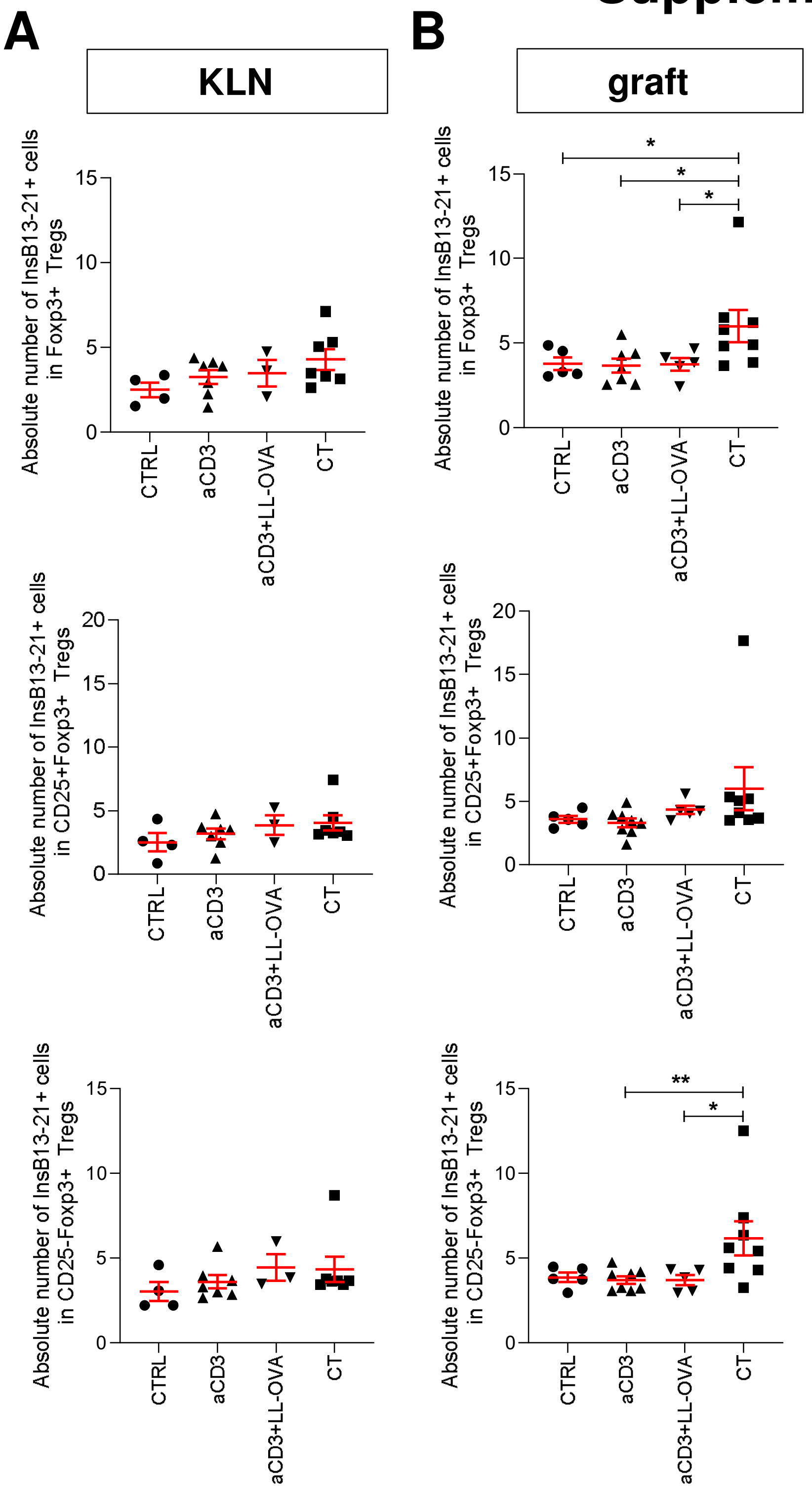

Supplementary Figure 8. Combination therapy increases numbers of InsB13–21$^+$Foxp3$^+$CD4$^+$ T cells in islet grafts. Newly diagnosed diabetic NOD mice with disease duration of less than 2 days were injected i.v. with alloxan (90 mg/kg) and transplanted with 500 syngeneic islets after 48 hours. Mice were left untreated (**CTRL**; n=4–5) or given a short-term low-dose aCD3 therapy (**aCD3**; n=7–8) either alone or combined with *L. lactis* bacteria secreting the irrelevant antigen ovalbumin (**LL-OVA**; n=3–5) or secreting beta cell antigen (PINS; n=7–8) combined with IL-10 (**CT**). Absolute numbers of tetramer positive (tet$^+$) InsB13–21 cells per 100 Foxp3$^+$ Tregs, either CD25$^+$ or CD25$^-$, are shown 3 weeks after islet substitution and therapy initiation in the kidney draining lymph nodes (KLN) (**A**) and islet grafts (**B**) of diabetic NOD mice. Symbols represent individual mice, and line and error bars reflect group mean ± SEM. Statistical significance between groups was calculated by Mann-Whitney U test; $*P < 0.05$, $**P < 0.01$.
